# Risk factors associated with prediabetes-to-diabetes conversion in a majority-minority county: a real-world analysis

**DOI:** 10.1007/s40200-026-01949-w

**Published:** 2026-05-13

**Authors:** Joyce Y. Lee, Ifigeneia Stathaki, Paul Gaura, Tiffany Huynh, Alexis Tran

**Affiliations:** 1 Department of Clinical Pharmacy Practice, School of Pharmacy and Pharmaceutical Sciences, University of California, Irvine, 802 W Peltason Dr (Berk Hall), Irvine, CA 92617 USA; 2 Albertsons Companies, Irvine, USA

**Keywords:** Prediabetes, Progression, Type 2 diabetes, Risk factors, Big data

## Abstract

**Purpose:**

To identify key risk factors associated with the progression from prediabetes to type 2 diabetes in a majority-minority county in the United States and evaluate their relative contributions.

**Methods:**

We conducted a retrospective cohort study using the deidentified EHR from the University of California, Irvine Health (2013–2023). Adults with prediabetes (confirmed by ICD codes and HbA1c values) were included, and those with diabetes of any form at baseline or with missing data were excluded. Baseline demographics and clinical factors were compared among those who progressed to T2D (converters) to those who did not (non-converters). Ordinal logistic regression and Cox proportional hazards models were used to assess predictors of conversion timing and risk.

**Results:**

A total of 2648 adults had confirmed prediabetes, of which 510 (19.3%) developed T2D. Among converters, 81.6% progressed within 5 years of the initial prediabetes diagnosis. The two groups were similar at baseline, except converters had higher obesity rates and were more likely to have Hispanic/Latino ethnicity (*p* < 0.001). Elevated baseline HbA1c was a strong independent predictor of earlier conversion to T2D (OR 0.41 per 1% increase, *p* < 0.001). Baseline triglycerides provided a modest additional effect (*p* = 0.047). Hispanic/Latino ethnicity was associated with a significantly higher risk of progression to T2D (adjusted HR 1.25, 95% CI 1.03–1.49, *p* = 0.021).

**Conclusions:**

Elevated HbA1c and triglycerides at baseline were associated with early conversion to T2D. This study underscores the need for early, targeted intervention in individuals with high-risk ethnic and clinical profiles to stop T2D.

## Introduction

Approximately 98 million Americans have prediabetes, and this is more than double the prevalence of diabetes in the United States [1]. Furthermore, once diagnosed, the detrimental impact of diabetes is economically burdensome, incurring medical expenditures that are 2.6 times higher than what would be expected without diabetes [2].

Lifestyle modification is the cornerstone of diabetes prevention and progression. However, active engagement in behavioral change among people with prediabetes is frequently hindered by the asymptomatic nature of prediabetes, which often presents with minimal to no overt clinical warning signs [3, 4]. Coupled with the busy lifestyle of many, epidemiological studies have reported that approximately 5–10% of those with prediabetes will progress to type 2 diabetes, and 70% will develop overt diabetes within their lifetime [5, 6].

While evidence-based programs such as the National Diabetes Prevention Program (DPP) [7] and the Prevent T2 Curriculum [8] and handouts are available, the number of new cases of type 2 diabetes continues to rise with up to 80% of people with prediabetes failing to follow up with any interventions, which include medical nutrition therapy, exercise, and metformin use in certain high-risk patients [9]. The suboptimal outcomes of prediabetes management are multifactorial, with a lack of tangible awareness and limited understanding of the long-term implications of dysglycemia among individuals with prediabetes being a major contributing factor [10, 11]. Additionally, time constraints due to increased patient volume among healthcare professionals limit their capacity to deliver personalized counseling and risk communications, further contributing to missed opportunities for timely intervention [12].

Another crucial, yet often overlooked, contributing factor is the underestimation of the clinical significance of a prediabetes diagnosis and its heterogeneous prognostic implications across socially and culturally diverse populations [13]. In a meta-analysis of 53 prospective cohort studies involving over 1 million individuals with a median follow-up duration of 9.5 years [14], prediabetes was significantly associated with increased incidence of cardiovascular outcomes, coronary heart disease, stroke, and chronic kidney disease. These findings suggest that pathophysiological changes and associated morbidity may begin during the prediabetes phase, reinforcing the urgency of early intervention.

Moreover, disparities in disease progression among ethnic and socioeconomic groups further complicate prevention efforts. The age-standardized prevalence of prediabetes is highest among Asians, followed by Hawaiians/Pacific Islanders and Hispanics [15]. Studies have shown that individuals from marginalized, racial and ethnic communities, including African American, Hispanic/Latino, and Native American populations, transition to type 2 diabetes more quickly than those of European descent [5, 16]. This rapid progression shortens the window for effective preventive intervention and underscores the need for culturally tailored strategies that address both clinical and social determinants of health. However, few studies have examined specific risk factors in heavily ethnic minority–populated communities, where intervention strategies may require specialized consideration.

Therefore, the primary objective of this study was to identify the key risk factors associated with progression from prediabetes to type 2 diabetes in a majority-minority county in Southern California. The secondary objective was to evaluate the relative contribution of these factors in the progression to diabetes conversion. By elucidating the predictive weight of individual and contextual variables, this study aimed to inform the development of more targeted and equitable prevention strategies for people with prediabetes.

## Research design & methods

### Study design and patient populations

We conducted a retrospective cohort study using a partially de-identified research copy of patient care records from the University of California, Irvine Health, structured according to the Observational Medical Outcomes Partnership (OMOP) Common Data Model. UC Irvine Health is located in Orange County, California, a majority-minority county (i.e., > 50% in terms of ethnic representation) with Hispanic/Latinx (H/L) and Asian Americans accounting for 35.0% and 21.1% of the population, respectively [17]. It is also the 6th most populous county in the United States. The dataset, obtained from the OMOP-based research data warehouse, includes patient health information spanning 10 years from January 1, 2013, to December 31, 2023.

Data were extracted by the UC Irvine Health Data Warehouse Analyst based on the preset criteria set by the study PI. These criteria included all patients with prediabetes, defined by the presence of ICD-10 diagnosis codes (R73.01, R73.02, R73.03) and laboratory-confirmed hemoglobin A1c (HbA1c) values in the range of 5.7% to 6.4% [18]. Patients were excluded if they had an ICD-10 diagnosis codes for type 1 or type 2 diabetes, a history of gestational diabetes, or evidence of off-label metformin use for the treatment of prediabetes throughout the 10-year study period. Subjects with a history of statin use were also excluded to reduce confounding from statins and statin-indicated comorbidities on the progression from prediabetes to diabetes [19].

This study was classified as non-human subjects research and did not require Institutional Review Board (IRB) approval; it was deemed exempt by the University of California, Irvine IRB (IRB#5315).

### Variables and outcomes

The independent variables of interest were baseline HbA1c, triglyceride (TG) levels, and body mass index (BMI), all measured at the time of initial prediabetes diagnosis (baseline). Demographic variables such as gender, race, and ethnicity were also included.

Covariates included age, sex, BMI, systolic blood pressure (SBP), diastolic blood pressure (DBP), race, ethnicity, baseline HbA1c, baseline TG, high-density lipoprotein (HDL), and low-density lipoprotein (LDL). These variables were selected based on the prior literature and their established clinical relevance to prediabetes progression [19].

The primary outcome was time (in days) from the initial prediabetes diagnosis to conversion to type 2 diabetes, as documented in the electronic health record. Conversion was defined by the ICD-10 diagnosis codes for type 2 diabetes and the type 2 diabetes lab criterion defined by the American Diabetes Association’s Standards of Medical Care in Diabetes.

### Statistical analysis

Baseline characteristics were summarized as mean ± standard deviation (SD) or as n (%) for categorical variables. Comparisons between converters (those who progressed to type 2 diabetes) and non-converters were made using two-sample *t* tests for continuous variables and χ² tests for categorical variables.

Conversion timing was described in five categories (< 1 year, 1–<2 years, 2–<3 years, 3–<5 years, > 5 years). To assess predictors of earlier versus later conversion, we fit an ordinal logistic regression model. We began with baseline variables and sequentially added TG, BMI, and ethnicity; we report odds ratios (ORs) with 95% confidence intervals (CIs). The proportional-odds assumption was verified with the Brant test. We compared candidate models using the Akaike Information Criterion (AIC) and retained the model with the lowest AIC [20]. To model actual time-to-conversion while accounting for right-censoring (participants who did not convert during observation or were lost to follow-up), we used Cox proportional hazards models. When the proportional hazards assumption was violated for HbA1c, we incorporated a time-varying covariate (HbA1c × log(time)). We report hazard ratios (HRs) with 95% CIs for the Cox models. For ethnicity, we conducted a series of Cox models with increasing adjustments: an unadjusted model, then models adding baseline HbA1c, then TG, then BMI, to examine how the association with Hispanic ethnicity changed with adjustment for these factors.

Missing data were handled by complete-case analysis. All tests were two-sided with α = 0.05. Analyses were conducted using Python.

## Results

### Patient demographics and rate of diabetes conversion

A total of 2,648 adults had confirmed prediabetes over a span of 10 years from January 1, 2013, to December 31, 2023. The majority of patients with prediabetes were white (1,582; 59.7%), female (1,584; 59.8%), with a baseline average age, BMI, and HbA1c of 57.6 ± 14.7 years, 30.0 ± 7.1 kg/m^2^, and 5.8 ± 0.5%, respectively (Table 1). The average baseline blood pressure, TG, and HDL levels were mostly within target, except for an elevated baseline average LDL of 112.2 ± 36.6 mg/dL.

**Table 1:** Baseline demographics and clinical characteristics of patients with prediabetes and by type 2 diabetes progression status

Overall cohort of Prediabetes (*N* = 2648)			Converter (*n* = 510)	Non-converter (*n* = 2138)	*p*-value
Demographics					
Age (years ± SD)		57.6 (14.7)	57.0 (14.4)	57.8 (14.8)	0.29
Gender (%)	Female	1584 (59.8)	289 (56.7)	1295 (60.6)	0.1307
	Male	1064 (40.2)	221 (43.3)	843 (39.4)	
Race (%)	White	1582 (59.7)	312 (61.2)	1270 (59.4)	0.7491
	Black or African American	102 (3.9)	19 (3.7)	83 (3.9)	
	Asian	417 (15.7)	73 (14.3)	344 (16.1)	
	American Indian/Alaskan Native	10 (0.4)	2 (0.4)	8 (0.4)	
	Native Hawaiian/Pacific Islander	17 (0.6)	4 (0.8)	13 (0.6)	
	Multiracial	74 (2.8)	25 (4.9)	49 (2.3)	
	Other Race	366 (13.8)	73 (14.3)	193 (3.6)	
	Unknown	80 (3.0)	2 (0.4)	78 (3.6)	
Ethnicity (%)	Hispanic or Latino	762 (28.8)	199 (39.0)	563 (26.3)	< 0.001
	Not Hispanic or not Latino	1790 (67.6)	307 (60.2)	1483 (69.4)	
	Uknown	96 (3.6)	4 (0.8)	92 (4.3)	
Clinical Characteristics					
BMI (kg/m^2^ ±SD)		30.0 (7.1)	31.9 (7.1)	29.6 (7.0)	< 0.001
BMI (%)	< 18.5	30 (1.1)	2 (0.4)	28 (1.3)	< 0.001
	19–24.9	586 (22.1)	76 (14.9)	510 (23.9)	
	25–29.9	822 (31.0)	137 (26.9)	685 (32.0)	
	30–34.9	675 (25.5)	135 (26.5)	540 (25.3)	
	35–39.9	322 (12.2)	98 (19.2)	224 (10.5)	
	> 40	213 (8.0)	62 (12.2)	151 (7.1)	
HbA1c (%±SD)		5.8 (0.5)	6.1 (0.7)	5.8 (0.4)	< 0.001
LDL (mg/dL ± SD)		112.2 (36.6)	107.8 (36.2)	113.3 (36.6)	0.002
TG (mg/dL ± SD)		137.1 (71.9)	148.0 (75.7)	134.5 (70.7)	< 0.001
HDL (mg/dL ± SD)		53.9 (16.6)	48.8 (14.1)	55.1 (16.9)	< 0.001
SBP (mg/dL ± SD)		128.6 (17.3)	129.3 (17.4)	128.6 (17.3)	0.37
SDP (mg/dL ± SD)		75.4 (11.1)	75.2 (11.2)	75.4 (11.1)	0.7

Footnote: Values are mean ± SD or n (%). *N* denotes total sample size; *n* denotes subgroup size. *BMI* body mass index, *HbA1c* hemoglobin A1c, *LDL* low-density lipoprotein cholesterol, *HDL* high-density lipoprotein cholesterol, *TG* triglycerides, *SBP* systolic blood pressure, *DBP* diastolic blood pressure

Among the patients with prediabetes, 510 (19.3%) converted to type 2 diabetes during the 10-year follow-up period, and 2138 (80.7%) remained in the prediabetes stage (non-converters). Among those who have progressed to type 2 diabetes (converters), 416 (81.6%) progressed to type 2 diabetes in less than 5 years (Table 2).

**Table 2:** Time to progression to type 2 diabetes among people with prediabetes (*n* = 510)

Time to Type 2 diabetes progression	Subjects with new onset of type 2 diabetes (%)
< 1 year	148 (29.0%)
1–2 years	101 (19.8%)
2–3 years	74 (14.5%)
3–5 years	93 (18.2%)

When comparing the converters and the non-converters, the baseline demographics and clinical characteristics were similar among the two groups, except HbA1c, ethnicity, BMI levels, and lipid panels (LDL, TG, and HDL), with more converters in the obesity category, self-identified as Hispanic/Latino and having higher baseline HbA1c and TG (*p* < 0.001) (Table 1). There were no significant differences in gender, race, or age between the converters and the non-converters.

### Baseline biomarkers and timing of progress to type 2 diabetes

**Fig. 1:**
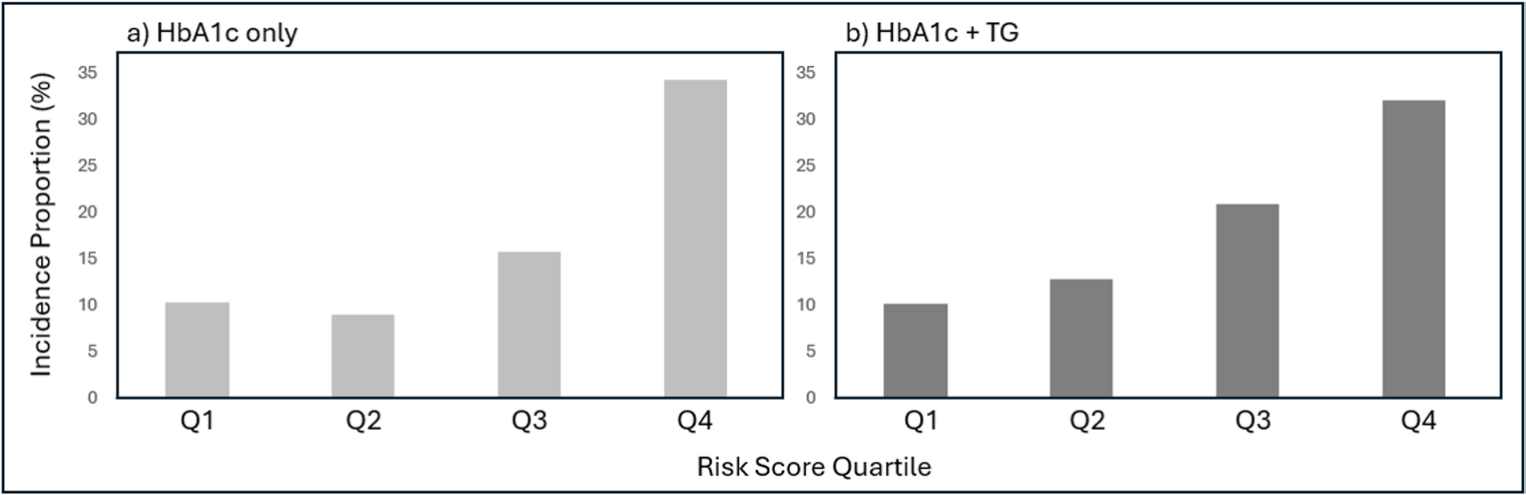
Conversion by (a) baseline HbA1c and (b) composite HbA1c and TG risk-score quartile. Footnote: *HbA1c* hemoglobin A1c, *TG* triglycerides

Using a series of ordinal logistic regression models, the average baseline HbA1c was associated with earlier conversion to type 2 diabetes (OR 0.41, 95% CI 0.29–0.58; *p* < 0.001), representing that each unit increase in the baseline HbA1c was associated with a 59% higher odds of converting earlier rather than later. Similarly, TG showed a small association (OR 0.998, 95% CI 0.996–1.000; *p* = 0.047). When we evaluated the association between a composite score using baseline TG and baseline A1C against the risk of conversion, we observed a proportionally increased risk of conversion across the quartiles of the composite scores (*p* < 0.001; Fig. 1). This was not observed with other clinical parameters, which included BMI, LDL, HDL, and blood pressure values.

### Ethnicity and conversion risk beyond baseline biomarkers

Although BMI and ethnicity variables significantly differed between converters and non-converters in univariate analysis, the effect of BMI was attenuated after adjusting for baseline A1C and TG in the multivariable model, while ethnicity remained significant (*p* = 0.006).

To further understand the role of ethnicity (Hispanic vs. non-Hispanic) in relation to diabetes progression, we compared baseline HbA1c levels between Hispanic and non-Hispanic individuals. In the overall population, Hispanic participants had significantly higher baseline HbA1c (5.9 ± 0.5% vs. 5.8 ± 0.4%;*p* < 0.001). Notably, this trend remained even when the analysis was restricted to individuals who converted to diabetes during follow-up.

An ethnicity-only Cox model showed that Hispanic participants had a 32% higher risk in progressing from prediabetes to type 2 diabetes compared to the Non-Hispanic participants (HR = 1.32, 95% CI: 1.10–1.58, *p* = 0.0029). The positive association between Hispanics ethnicity and the risk of conversion remains statistically significant after adjustment for baseline HbA1c, TG and BMI (fully-adjusted HR = 1.25, 95% CI: 1.03–1.49, *p* = 0.021) (Table 3).

**Table 3:** Association between hispanic ethnicity and risk of diabetes conversion in progressively adjusted cox model

Adjustments Added	HR (Hispanic vs. Non-Hispanic)	95% CI	*P*-value
Ethnicity only	1.32	1.10–1.58	0.0029
+ HbA1c	1.28	1.07–1.53	0.0075
+ HbA1c and TG	1.26	1.05–1.50	0.0131
+ HbA1c, TG, and BMI	1.25	1.03–1.49	0.0210

*BMI* Body Mass Index, *HbA1c* hemoglobin A1c, *TG* Triglycerides, *HR *Hazard Ratio

## Discussion

In this real-world study, we leveraged a robust, de-identified electronic health record database from a public university health system in a majority-minority county in California, enabling longitudinal tracking of individuals with prediabetes over a 10-year period. This comprehensive dataset facilitated an examination of the natural history of prediabetes and its progression to type 2 diabetes within a real-world clinical population. Our findings confirmed that HbA1c, especially the elevated levels of HbA1c, was a significant predictor of conversion to type 2 diabetes. Moreover, elevated triglyceride levels, in addition to increasing HbA1c, also increased the risk of progression to type 2 diabetes, further amplifying the predictive value of HbA1c. Importantly, our analysis also identified the significance of Hispanic ethnicity as an independent risk factor for progression from prediabetes to type 2 diabetes. Findings from this study underscore the urgent need for targeted prediabetes management strategies, particularly among specific ethnic groups at increased risk for type 2 diabetes.

In our study, the overall 10-year conversion rate from prediabetes to type 2 diabetes among our cohort was 19.3%, which is notably lower than the conversion rates reported in the broader literature. Previous longitudinal studies have estimated that up to 70% of individuals with prediabetes may progress to type 2 diabetes over 10 years if left untreated [5, 6]. This discrepancy may be attributed in part to the demographic composition of our study population, which predominantly consisted of non-Hispanic White individuals, historically associated with a lower risk of progression compared to certain racial and ethnic minorities [16, 18, 20].

The Centers for Disease Control and Prevention has reported that the prevalence of type 2 diabetes is nearly twice as high in Hispanic adults compared to non-Hispanic White adults (11.8% vs. 6.9%) [1]. While genetic predisposition and insulin resistance are well-established contributors to this disparity, cultural, dietary, and environmental factors also play a significant role in the increased risk observed in Hispanic populations [21, 22]. Our sub-analysis further supports these trends, where among individuals who progressed to type 2 diabetes, over half were White Hispanic subjects. Notably, the conversion rate of these subjects was 29.0% during the first year of prediabetes diagnosis, markedly higher than the general population estimates of 5–10% [5, 6]. These findings reinforce the need for tailored prevention strategies targeting high-risk populations. A uniform, one-size-fits-all approach to prediabetes management may not be sufficient to reduce the burden of new-onset type 2 diabetes, particularly in communities with high-risk profiles.

In our study, HbA1c, particularly at higher levels at the time of prediabetes diagnosis, was confirmed to be a strong predictor of progression to type 2 diabetes. Although clinical parameters such as HbA1c, BMI, and triglyceride levels are conventionally linked to type 2 diabetes due to their strong associations with lifestyle and metabolic factors [23], our findings indicated that triglyceride concentrations near the upper limit of the normal range (mean triglyceride: 148 mg/dL) can substantially enhance the effect of elevated HbA1c, signaling an imminent transition from prediabetes to type 2 diabetes. A meta-analysis of 61 prospective studies [24] confirmed that while triglyceride levels below 90 mg/dL were not significantly associated with cardiovascular disease or all-cause mortality, individuals with intermediate triglyceride levels (90–149 mg/dL) still exhibited an increased risk, which became progressively more pronounced at higher concentrations. Given the close interrelationship among triglyceride levels, dietary intake, insulin resistance, and glycemic control, our results suggested that triglyceride levels around 148 mg/dL, though considered within the conventional normal range, may nonetheless warrant stricter metabolic monitoring and management, particularly in individuals with elevated HbA1c.

This study has several limitations. First, independent variables were assessed only at baseline, without consideration of longitudinal changes over time, which may have provided a more accurate depiction of individual risk trajectories. Second, although key covariates were included in the analyses, residual confounding cannot be excluded, as important sociodemographic and lifestyle factors such as family history, dietary habits, physical activity, and socioeconomic status were not available. Third, only HbA1c was used to confirm prediabetes status when it was first documented through ICD-10 codes. Although HbA1c is widely used for screening in outpatient settings, the lack of use of other diagnostic measures, such as fasting plasma glucose, oral glucose tolerance testing, or random plasma glucose, may have resulted in under-identification of individuals with prediabetes. Finally, because the data were derived from a single university health system in Southern California, the generalizability of these findings may be limited to similar regions with similar heterogeneous patient populations.

## Conclusion

Hispanic/Latino ethnicity was an independent predictor of early conversion to type 2 diabetes in this study that was conducted in a majority-minority county. Elevated HbA1c at the time of prediabetes diagnosis was also an independent predictor and the presence of elevated triglyceride further amplified the impact of HbA1c on the early conversion to type 2 diabetes. Prediabetes management in majority-minority population should be strategic, targeting the high-risk ethnic populations and those with clinical factors that make them more prone to progression to type 2 diabetes.

## Data Availability

No datasets were generated or analysed during the current study.
